# Is radical radiotherapy with/without surgery an effective treatment in the lymphoepithelial carcinoma of the salivary gland？

**DOI:** 10.1186/s12885-023-11466-1

**Published:** 2023-10-12

**Authors:** Xiaoshuang Niu, Peiyao Liu, Xiaoshen Wang, Chaosu Hu

**Affiliations:** 1 Department of Radiation Oncology, Fudan University Shanghai Cancer Center, Shanghai, China; 2 Department of Oncology, Shanghai Medical College, Fudan University, Shanghai, 200032 China; 3 Department of Radiation Oncology, Shanghai Clinical Research Center for Radiation Oncology, Shanghai, China; 4 Shanghai Key Laboratory of Radiation Oncology, Shanghai, China; 5 Department of Radiation Oncology, Eye & ENT Hospital, Fudan University, Shanghai, China

**Keywords:** Lymphoepithelial carcinoma, Salivary gland, Radical radiotherapy, Surgery, Effective treatment

## Abstract

**Background:**

There is limited information of radical radiotherapy (RT) on lymphoepithelial carcinoma of salivary gland (LECSG) regarding to the rarity of the disease. We conducted this retrospective study that evaluated the feasibility and efficacy of radical RT with/without surgery in LECSG.

**Methods:**

We retrospectively reviewed patients that were pathologically diagnosed of LECSG and had definite or suspicious residual disease. The prescribed dose given to P-GTV and/or P-GTV-LN was 66 to 70.4 Gy. The clinical target volume (CTV) involved ipsilateral salivary gland and corresponding lymph node drainage area.

**Results:**

A total of 56 patients were included. With a median follow-up of 60 months (range: 8 to 151 months), the 1-, 5-, and 10-year progression-free survival (PFS) rates were 94.6%, 84.7% and 84.7%; locoregional progression-free survival (LRPFS) rates were 98.2%, 87.4% and 87.4%; distance metastasis-free survival (DMFS) rates were 94.6%, 86.7% and 86.7%; and overall survival (OS) rates were 98.2%, 92.4% and 89.0%, respectively. A total of 7 patients without surgery were included. All patients were alive and only one patient experienced failure of distant metastasis four months after RT. The results of univariate analysis showed that compared with N stage, the number of positive lymph nodes (2 positive lymph nodes) was better prognostic predictor especially in PFS. There were no treatment-related deaths and most toxicities of RT were mild.

**Conclusions:**

Radical RT with/without surgery in LECSG for definite or suspicious residual disease is feasibility and efficacy. Most toxicities of RT were mild due to the target volume involved ipsilateral area.

## Introduction

Lymphoepithelial carcinoma (LEC) is a rare malignant tumor, which was originally described by Hilderman in 1962 [1]. LEC occurs in multiple sites such as the salivary glands, oral cavity, lung, stomach, urinary tract and so on [2, 3]. It is characterized with an undifferentiated nonkeratinizing squamous cell carcinoma accompanied by a prominent non-neoplastic lymphoplasmacytic infiltration, which is identical to undifferentiated nasopharyngeal carcinoma (NPC) [4]. Many studies have pointed out Epstein-Barr virus (EBV) infection is a contributing factor to the occurrence and development of the disease as well as sensitivity to irradiation similar to NPC [5–8].

The major salivary glands are the most common sites and more than 80% occurs in the parotid gland [3, 9–11]. Surgery has been considered the most important treatment of LEC of salivary gland (LECSG) according to the NCCN Guidelines for Head and Neck Cancers [12]. However, it is often difficult to remove the tumor completely of LECSG due to the facial nerves protection. To the patients’ quality of life, radiotherapy (RT) is supposed to be a vital treatment. There is limited information of evaluating radical RT on patient outcome regarding to the rarity of the disease and a dearth of prospective data. Here, we conducted this retrospective study that evaluated the feasibility and efficacy of radical RT with/without surgery in LECSG.

## Materials and methods

### Patients and diagnosis

This was a retrospective study that evaluated the feasibility and efficacy of radical RT with/without surgery in LECSG. All patients were pathologically diagnosed by core needle aspiration or surgery. All patients had definite or suspicious residual disease and received dose of RT ≥ 66 Gy. They were all re-staged by the 8th American Joint Committee on Cancer (AJCC) criteria. The patients were staged by pathological (surgical) if they had surgery, otherwise by image combined with clinical features.

It was difficult to make an accurate diagnosis of LECSG before treatment because there were no typical symptoms or examinations. Initial assessment consisted of medical history and physical examination, enhanced MRI/CT of the salivary gland and/or cervical, ultrasound or lesion site fine needle aspiration biopsy (FNAB). Other assessment included positron emission tomography-CT (PET-CT), or replaced by chest CT, abdominal ultrasound/CT and bone emission CT. To make a correct diagnosis and exclusion of NPC, physical examination of nasopharyngeal combined with nasopharyngoscopy and imaging (CT, MR) examinations was conducted before RT.

### Treatment

Before RT, all patients were therapied by core needle aspiration, salivary gland disease biopsy, resection of the primary location alone or resection of both the primary location and cervical lymph nodes. 3D conformal RT (3D-CRT) or intensity-modulated RT (IMRT) was administrated in our hospital for all patients. Platinum-based chemotherapy was used for patients with high risk factors.

The gross tumor volume (GTV) included the region of positive margin and residual salivary tumor/cervical lymph nodes, confirmed by physical examination, enhanced MRI/CT and/or PET-CT. The clinical target volume (CTV) was considered as subclinical lesions that included the whole involved ipsilateral salivary gland and corresponding lymph node drainage area. The planning target volume (PTV) would consist of the GTV/CTV with a 3–5 mm margin. The prescribed dose given to P-GTV (the region of positive margin and residual salivary tumor) and/or P-GTV-LN (residual positive cervical lymph nodes) were 66 to 70.4 Gy. The PTV covering the high-risk CTV was 57–60 Gy. The PTV covering the low-risk CTV was 50-54.4 Gy.

### Assessment and follow-up

Radiotherapy-related toxicities were assessed according to the Radiation Therapy Oncology Group (RTOG) and determined by a retrospective chart review. Patients were assessed weekly during RT to monitor the treatment response and toxicity. After treatment completion, follow-ups were repeated every 3 months for the first 2 years, every 6 months from the third through the fifth year and annually thereafter. Routine follow-up included medical history and physical examination. Enhanced MRI/CT or ultrasonography of the primary site was performed in the routine follow-up. Chest CT and ultrasonography of the abdomen were conducted once yearly. Bone emission CT was performed when there were clinical indications.

### Statistical analysis

SPSS 23.0 (SPSS Inc, Chicago, IL, USA) was used for statistical analysis in this study. The factors of categorical variables were compared with the chi-square test or Fisher exact test. Overall survival (OS) was calculated from the date of diagnosis to the date of death for any cause or last follow-up. Progression-free survival (PFS) was calculated from the date of diagnosis to the date of tumor progression, death for any cause or last follow-up. Locoregional progression-free survival (LRPFS) was calculated from the date of diagnosis to the date of locoregional failure or the date of death or last follow-up. Distance metastasis-free survival (DMFS) was calculated from the date of diagnosis to the date of metastasis or the date of death or last follow-up. The OS, PFS, LRPFS and DMFS were calculated by the Kaplan–Meier method. Log-rank test was used to examine the differences between groups. A 2-sided P < 0.05 was considered statistically significant.

The method descriptions could be referred to our previously published article (Niu, X., et al., Is postoperative radiotherapy an essential treatment for nonmetastatic lymphoepithelial carcinoma of the salivary gland? Radiother Oncol, 2022. 172: p. 76–82.)

## Results

### Patients

From Jan 2008 to December 2020, 56 patients pathologically diagnosed with LECSG by core needle aspiration or surgery were enrolled in this study. All patients had definite or suspicious residual disease and received dose of RT ≥ 66 Gy. The median dose of PTV-G was 66 Gy (range, 66–70.4 Gy). FNAB was performed preoperatively in 17 patients, and 15 of 17 patients (92.9%) presented with a malignant tumor. They were all re-staged by the 8th AJCC criteria and one patient was M1 (multiple bone metastases) at the initial diagnosis. Platinum-based chemotherapy was used for 20 patients with high risk factors. 13 patients received concurrent chemotherapy and 13 patients received induction or adjuvant chemotherapy. The characteristics of patients and detailed features of treatment were shown in Table 1.

**Table 1:** Characteristics of patients

Characteristic	No. of patients	Percent(%)
Age (years)		
Median 38.5 Range 15–86		
≤ 38	28	50.0
＞38	28	50.0
Gender		
Male	29	51.8
Female	27	48.2
Primary site		
Parotid gland	48	85.7
Submandibular gland	8	14.3
T Stage		
T1	12	21.4
T2	34	60.7
T3	8	14.3
T4	2	3.6
Maximum diameter of primary lesion (cm)		
≤ 3	36	64.3
> 3	20	35.7
N Stage		
N0	21	37.5
N1	10	17.9
N2	21	37.5
N3	4	7.1
Number of positive lymph nodes		
≤ 2	36	64.3
> 2	20	35.7
Total Stage		
I	8	14.3
II	10	17.9
III	12	21.4
IV	26	46.4
Treatment		
core needle aspiration	4	7.1
salivary gland disease biopsy	3	5.4
resection of the primary location	38	67.9
resection of both the primary location and cervical lymph nodes	11	19.6
Chemotherapy		
No	36	64.3
Yes	20	35.7

### Total survival outcomes

**Fig. 1:**
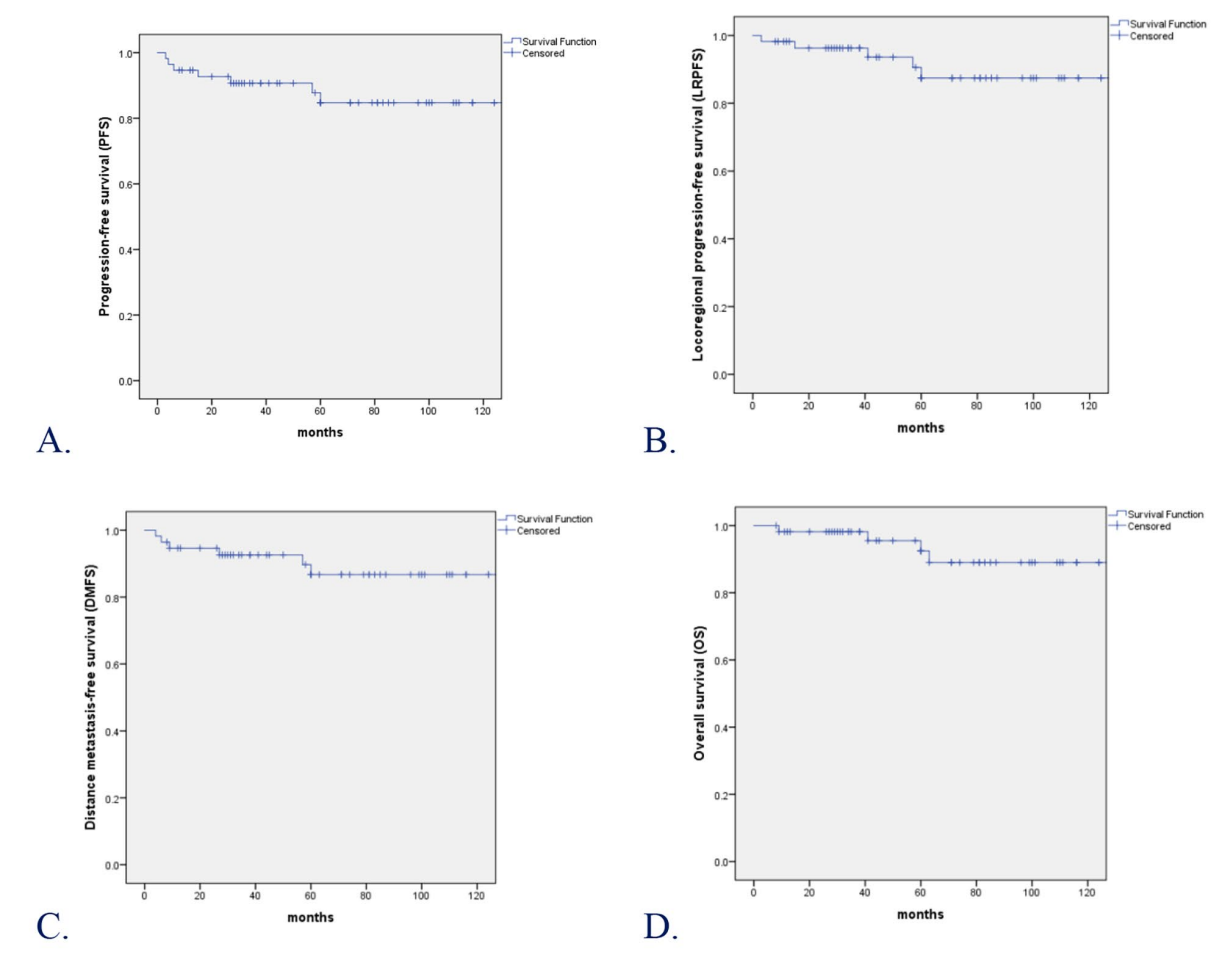
Kaplan–Meier estimate of **(A)** PFS, **(B)** LRPFS, **(C)** DMFS, and **(D)** OS curves for all the patients

With a median follow-up of 60 months (range: 8 to 151 months), 52 of 56 patients (92.9%) were alive, 2 (3.6%) developed locoregional failure, 4 (7.1%) experienced distant metastasis, 7 (12.5%) developed tumor progression or death and 1 (1.8%) had the second primary tumor. At the last follow-up visit, a total of 4 patients died: 3 patients died of distant metastasis and 1 of other disease. For the whole cohort, the 1-, 3-, 5-, and 10-year PFS rates were 94.6%, 90.7%, 84.7% and 84.7%, respectively; LRPFS rates were 98.2%, 96.2%. 87.4% and 87.4%, respectively; DMFS rates were 94.6%, 92.6%, 86.7% and 86.7%, respectively; and OS rates were 98.2%, 98.2%, 92.4% and 89.0%, respectively (Fig. 1).

### Survival outcomes without surgery

A total of 7 patients without surgery were included in the study: 4 patients with core needle aspiration and 3 patients with salivary gland disease biopsy. Of them, one patient was multiple bone metastases and six with positive lymph nodes of neck. 5 of 7 patients (71.4%) presented with stage IV. 5 (71.4%) patients received chemotherapy, consisting of 3 with induction chemotherapy (IC) and 4 with concurrent chemotherapy (CCRT). The median follow-up was 60 months (range: 11 to 151 months), all patients were alive and only one patient experienced failure of distant metastasis four months after RT.

**Fig. 2:**
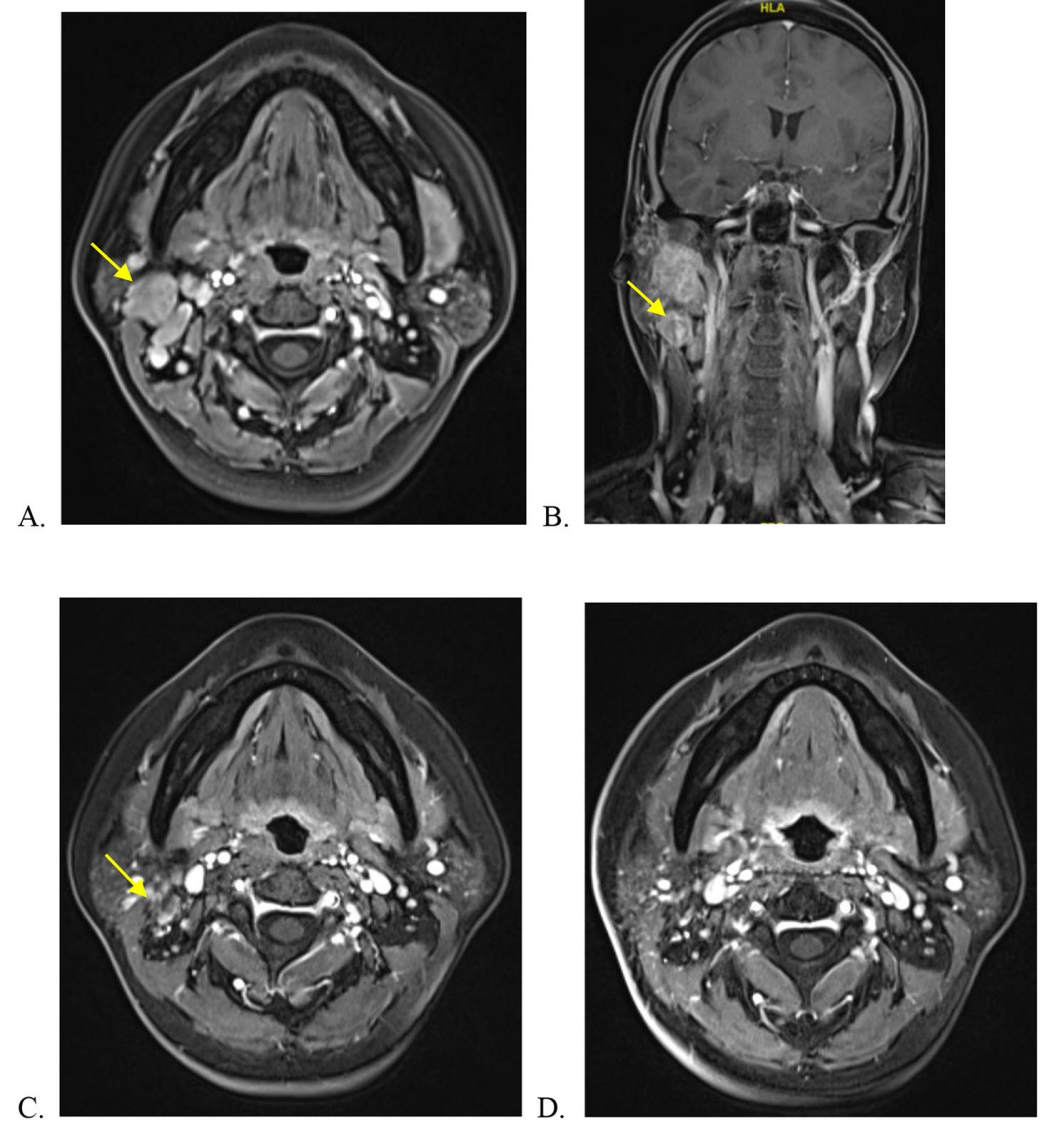
**(A)** The primary tumor was detected in the right parotid; **(B)** the enlarged lymph nodes in ipsilateral side before the treatment; **(C)** after IC; **(D)** after CCRT

A typical case was shown as following. A 38-year-old woman complained of a palpable and slowly growing mass in the right parotid area for 3 months. Enhanced MRI of the salivary gland, CT of cervical and PET-CT revealed a solid tumor mass located across the deep lobe of the parotid gland and enlarged lymph nodes in ipsilateral levels II and III. She was pathologically diagnosed by core needle aspiration and in situ hybridization for EBV-encoded small RNAs (EBERs) was positive in the tumor cells. The quantification of EBV-DNA load was 6.17*10^2^ copy/mL. Examination of the nasopharynx found no lesions. Based on the clinical, radiological and pathological, a final diagnosis (T2N2bM0 IVA, AJCC 8th) was made. The patient was treated with IC and CCRT. The IC regimen was a combination of 75 mg/m^2^ docetaxel and 75 mg/m^2^ nedaplatin q3w. CCRT consisted of nedaplatin 75 mg/m^2^ q3w. The radiation dose schedule was as follows: the planning gross tumor volume (P-GTV and P-GTV-LN) was prescribed a dose of 70.4 Gy; PCTV1, 57.6 Gy; and PCTV2, 54.4 Gy. A partial response (PR) was achieved after IC and complete response (CR) was achieved after CCRT. No serious adverse events were found. There was no evidence of recurrence or metastasis after 40 months (Fig. 2).

### Prognostic analysis

Impact of prognostic factors on PFS, LRPFS, DMFS and OS were evaluated using univariate analyse, including age at diagnosis, gender, primary site, T stage, maximum diameter of primary lesion, N stage and number of positive lymph nodes. (Table 2) Age (P = 0.048) and T stage (P = 0.041) were the two factors that significantly influenced OS. Number of positive lymph nodes (≤ 2) (P = 0.027) appeared to be positive prognostic factor for PFS. Gender (P = 0.037) was the factor that significantly influenced LRPFS. The results of univariate analysis showed that compared with N stage, the number of positive lymph nodes (2 positive lymph nodes) was better prognostic predictor especially in PFS.

### Radiation toxicities

There were no treatment-related deaths. Oral mucositis and skin reactions were the most common acute treatment toxicities in our study. Among all the patients, 31 (55.4%), 15 (26.8%) and 5 (8.9%) had grade 1, 2 and 3 mucositis, respectively. Most skin reactions were grades 0 to 2. A total of 5 patients suffered grade 3 skin toxicity. Overall, most late injuries were assessed as grades 0 to 1. The most common late toxicity was neck fibrosis, which occurred in five patients who underwent both radical neck dissection and whole neck irradiation. One patient had the second primary tumor, of whom was lymphoma.

**Table 2:** Univariate analysis of prognostic factors

Characteristic	N (%)	5- year PFS (%)	P value	5-year LRPFS (%)	P value	5-year DMFS (%)	P value	5-year OS (%)	P value
Age									
≤ 38	28 (50.0)	92.4	0.241	96.0	0.178	96.5	0.090	100.0	**0.048**
> 38	28 (50.0)	77.0		79.2		77.0		85.3	
Gender									
Male	29 (51.8)	78.5	0.320	77.7	**0.037**	82.4	0.535	86.5	0.068
Female	27 (48.2)	92.6		100.0		92.6		100.0	
Primary site									
Parotid gland	48 (85.7)	87.8	0.255	89.2	0.637	90.1	0.146	94.9	0.296
Submandibular gland	8 (14.3)	58.3		66.7		58.3		66.7	
T stage									
1–2	46 (82.1)	87.2	0.382	90.7	0.131	89.6	0.266	93.1	**0.041**
3–4	10 (17.9)	72.0		72.0		71.1		88.9	
maximum diameter of primary lesion									
≤ 3 cm	36 (64.3)	86.8	0.597	91.2	0.185	86.8	0.934	91.2	0.498
> 3 cm	20 (35.7)	80.2		80.2		86.1		94.7	
N stage									
0–1	31 (55.4)	88.5	0.117	88.5	0.416	88.5	0.251	94.1	0.791
2–3	25 (44.6)	78.7		84.7		83.2		89.4	
Number of positive lymph nodes									
≤ 2	36 (64.3)	90.2	**0.027**	90.2	0.191	90.2	0.089	95.0	0.498
> 2	20 (35.7)	72.9		80.6		78.7		86.8	

## Discussion

We retrospectively evaluated the feasibility and efficacy of radical RT with/without surgery in LECSG. Although surgery has been considered the standard treatment of LECSG, it is often difficult to remove the tumor completely of LECSG due to the facial nerves protection. Owing to the histologically indistinguishable from NPC, the relationship with EBV and the predilection of both cancers for certain regions and populations, we assume that LECSG is sensitivity to RT as NPC. Meantime, to the patients’ quality of life, RT is supposed to be a vital treatment to the patients’ quality of life.

In our study, all patients had definite or suspicious residual disease and received dose of RT ≥ 66 Gy. For the whole cohort, the 1-, 3-, 5-, and 10-year PFS rates were 94.6%, 90.7%, 84.7% and 84.7%, respectively; LRPFS rates were 98.2%, 96.2%. 87.4% and 87.4%, respectively; DMFS rates were 94.6%, 92.6%, 86.7% and 86.7%, respectively; and OS rates were 98.2%, 98.2%, 92.4% and 89.0%, respectively. The results of 3-year LRPFS and OS were higher than the rates reported in the treatment of definitive surgical resection and postoperative RT. The rates of 3-year locoregional control (LRC) and OS in previous studies [13] in Fudan University Shanghai Cancer Center were 94.3% and 92.9%, respectively. The results of our research were also higher than the rates reported in the non-endemic region. The 5- and 10- year OS rates in a retrospective review of the National Cancer Database (NCDB) from 1998 to 2012 by Zhan et al. [14] were 77.0% and 56.0%, respectively. RT was appropriate initial locoregional therapy for patients with non-NPC lymphoepithelioma and surgery should be reserved for patients who had persistent disease after RT [15]. LECSG has a high propensity nodal metastasis with 58.3% at diagnosis [16], IC + CCRT for advanced stage LECSG might be a reasonable approach [17].

The treatment failure of our research was that only 2 (3.6%) patients developed locoregional failure and no patient died of it. The critical factor might be that the dose of RT was sufficient and all patients received dose of RT ≥ 66 Gy. RT is one of the most important means to improve locoregional control and protect the organ at risk of LECSG, as the similar conclusion was found in Zhan’s study [14]. Wang and colleagues [18] found that postoperative RT could improve long-term survival owing to decreasing the risk of recurrence among those patients. Ma’s study [19] also reported that postoperative RT was associated independently with relapse-free survival. Meanwhile, most toxicities of RT were mild due to the target volume involved ipsilateral area. Oral mucositis and skin reactions were the most common acute toxicities in our study and most reactions were grades 0 to 2.

In our study, the results of univariate analysis showed that compared with N stage, the number of positive lymph nodes (2 positive lymph nodes) was better prognostic predictor especially in PFS. Though the limitation of our study was a retrospective and relatively small sample study, optimizing the tumor-node-metastasis (TNM) staging system of salivary gland might be necessary. In addition, there was lack of information on the EBV status in our cohort. A high prevalence of EBV infection may contribute to the development of LECSG. Determination of EBV status is valuable in diagnosing LECSG and judging the effect of treatment [5–8]. The patients were enrolled in our study from Jan 2008 to December 2020. The research has a large time span, and the test of EBV-DNA has developed in recent years in our hospital.

## Conclusions

Radical RT with/without surgery in LECSG for definite or suspicious residual disease is feasibility and efficacy. The dose of all patients received RT ≥ 66 Gy. The locoregional failure was low and no patient died of it. The results of univariate analysis showed that compared with N stage, the number of positive lymph nodes (2 positive lymph nodes) was better prognostic predictor especially in PFS. Most toxicities of RT were mild due to the target volume involved ipsilateral area. Limitations of the study such as retrospective data, long time span and single center should be considered in the future research.

## Data Availability

Data will be made available on reasonable request and you can contact Xiaoshuang Niu (mail address: xniu13@fudan.edu.cn).

## Abbreviations

RT: radical radiotherapy
LECSG: lymphoepithelial carcinoma of salivary gland
CTV: clinical target volume
PFS: progression-free survival
LRPFS: Standard
SGML: locoregional progression-free survival
DMFS: distance metastasis-free survival
OS: overall survival
LEC: Lymphoepithelial carcinoma
NPC: nasopharyngeal carcinoma
EBV: Epstein-Barr virus
AJCC: American Joint Committee on Cancer
FNAB: fine needle aspiration biopsy
PET-CT: positron emission tomography-CT
3D-CRT: 3D conformal RT
IMRT: intensity-modulated RT
GTV: gross tumor volume
PTV: planning target volume
RTOG: Radiation Therapy Oncology Group
IC: induction chemotherapy
CCRT: concurrent chemotherapy
EBERs: EBV-encoded small RNAs
PR: partial response
CR: complete response
LRC: locoregional control
NCDB: National Cancer Database
TNM: tumor-node-metastasis

## References

[1] HILDERMAN W C, GORDON J S, LARGE H J (1962). Malignant lymphoepithelial lesion with carcinomatous component apparently arising in parotid gland. A malignant counterpart of benign lymphoepithelial lesion?[J]. Cancer.

[2] Whelan A, Al-Sayed AA, Bullock M (2020). Primary parotid lymphoepithelial carcinoma: a case report and literature review of a rare pathological entity[J]. Int J Surg Case Rep.

[3] Gupta S, Loh KS, Petersson F (2012). Lymphoepithelial carcinoma of the parotid gland arising in an intraglandular lymph node: report of a rare case mimicking metastasis[J]. Ann Diagn Pathol.

[4] Barnes L, Eveson JW, Reichart P (2005). World health organization classification of tumours: pathology and genetics of head and neck tumours [M].

[5] Maeda H, Yamashiro T, Yamashita Y (2018). Lymphoepithelial carcinoma in parotid gland related to EBV infection: a case report[J]. Auris Nasus Larynx.

[6] Ambrosio MR, Mastrogiulio MG, Barone A (2013). Lymphoepithelial-like carcinoma of the parotid gland: a case report and a brief review of the western literature[J]. Diagn Pathol.

[7] Saku T, Cheng J, Jen KY (2003). Epstein-Barr virus infected lymphoepithelial carcinomas of the salivary gland in the Russia-Asia area: a clinicopathologic study of 160 cases[J]. Arkh Patol.

[8] Jen KY, Cheng J, Li J (2003). Mutational events in LMP1 gene of Epstein-Barr virus in salivary gland lymphoepithelial carcinomas[J]. Int J Cancer.

[9] Manganaris A, Patakiouta F, Xirou P (2007). Lymphoepithelial carcinoma of the parotid gland: is an association with Epstein-Barr virus possible in non-endemic areas?[J]. Int J Oral Maxillofac Surg.

[10] Picon H, Guddati AK (2021). Analysis of Trends in Mortality in patients with Lymphoepithelial Carcinoma of the Head and Neck[J]. Int J Gen Med.

[11] Thompson L, Whaley RD (2021). Lymphoepithelial Carcinoma of Salivary Glands[J]. Surg Pathol Clin.

[12] Pfister DG, Spencer S, Adelstein D (2020). Head and Neck Cancers, Version 2.2020, NCCN Clinical Practice Guidelines in Oncology[J]. J Natl Compr Canc Netw.

[13] Li F, Zhu G, Wang Y (2014). A clinical analysis of 37 cases with lymphoepithelial carcinoma of the major salivary gland treated by surgical resection and postoperative radiotherapy: a single institution study[J]. Med Oncol.

[14] Zhan KY, Nicolli EA, Khaja SF (2016). Lymphoepithelial carcinoma of the major salivary glands: predictors of survival in a non-endemic region[J]. Oral Oncol.

[15] Dubey P, Ha CS, Ang KK (1998). Nonnasopharyngeal lymphoepithelioma of the head and neck[J]. Cancer.

[16] Yin L, Huang X, Liu X (2017). Distribution of lymph node metastasis from lymphoepithelial-like carcinoma of the parotid[J]. Oncotarget.

[17] Lv S, Xie D, Wu Z et al. Is surgery an Inevitable treatment for Advanced Salivary Lymphoepithelial Carcinoma? Three Case Reports[J]. Ear Nose Throat J, 2020:584294434. 10.1177/0145561320923170 32380853

[18] Wang JQ, Deng RX, Liu H (2020). Clinicopathological characteristics and prognostic analysis of lymphoepithelial carcinoma of salivary gland: a population-based study[J]. Gland Surg.

[19] Ma H, Lin Y, Wang L (2014). Primary lymphoepithelioma- like carcinoma of salivary gland: sixty-nine cases with long- term follow-up. Head Neck.

